# What predicts word reading in Arabic?

**DOI:** 10.3389/fpsyg.2023.1077643

**Published:** 2023-04-27

**Authors:** Elsayed E. A. Hassanein, Evelyn S. Johnson, Sayed Ibrahim, Yousef Alshaboul

**Affiliations:** ^1^Department of Psychological Sciences, College of Education, Qatar University, Doha, Qatar; ^2^Department of Early and Special Education, Boise State University, Boise, ID, United States; ^3^Department of Educational Sciences, College of Education, Qatar University, Doha, Qatar

**Keywords:** Arabic, phonological processing, morphological processing, orthographic processing, word reading

## Abstract

Efficient and accurate word reading ability is critical for later reading success. As such, it is important to understand the component skills that underlie strong word reading ability. Although a growing research base points to the importance of phonological processing, morphological processing and orthographic processing for accurate and fluent word reading in Arabic, there are few studies that examine all three areas at one time to better understand their role in word reading. Additionally, it remains unclear whether the contribution of the various processes might differ across the early years when children are learning to read. 1,098 pupils in grades 1–3 participated in this study and took tests for phonological processing, morphological processing, orthographic processing, and word reading accuracy and fluency. According to the findings of regression analyses, the relative contribution of these underlying processes differed according to the method used to test word reading and the student’s grade level. Regarding accuracy, several subscales of phonological processing and two measures of orthographic processing accounted for significant differences in word reading accuracy for first graders. For second grade students, nonword repetition, elision, and all three measures of orthographic processing accounted for variance. In third grade, elision and memory for digits, word creation and morpheme identification, and letter/sound identification and orthographic fluency were significant predictors of word reading accuracy. In terms of fluency, two subscales of phonological processing, two measures of orthographic processing, and two measures of morphological processing explained significant differences in word reading fluency for first graders. For second grade students, nonword repetition, elision, RAN-digits, isolation, segmenting and all the measures of orthographic processing and word creation explained unique variance in word reading fluency. In third grade, elision, RAN-letters, RAN-digits and phoneme isolation, all measures of orthographic processing and morphological processing, explained variance in word reading fluency. Implications and future directions in research are discussed.

## 1. Introduction

Efficient and accurate word reading plays a critical role in early reading development (Castles et al., 2018). Difficulty reading words accurately and fluently in isolation is one of the most consistent predictors of later reading problems (Steacy et al., 2022). Therefore, beginning readers must develop the skills needed to access the words on the page to learn to read well. According to the Reading Systems Framework (Perfetti and Stafura, 2014), there are several processes that must be integrated to facilitate efficient word identification. These include phonological processing, morphological processing and orthographic processing.

The Reading Systems Framework (Perfetti and Stafura, 2014) presents a universal model that reflects the common, cognitive operations involved in reading, regardless of the various writing systems used (Frost, 2012). In this model, visual input (orthography) is mapped to the corresponding phonological units to allow for the representation and identification of words. Word meaning is supported through the mental lexicon, that draws on knowledge of morphology and syntax to select the relevant, specific word meaning. Comprehension is facilitated through word identification, and both oral language and general background knowledge are activated as the reader interacts with the text. From this model, Perfetti derived the Lexical Quality Hypothesis, which posits that the quality of the reader’s knowledge of a given word is the bridge between decoding and comprehension (Perfetti, 2007). When a reader has deep knowledge of a word, it supports rapid and efficient word identification, which then frees attentional resources for higher-level processing needs, such as comprehension (Perfetti and Stafura, 2014). This suggests that: (1) comprehension depends on successful word reading, and (2) skill differences in comprehension can arise from skill differences in word reading (Perfetti and Stafura, 2014).

There is strong research support for the Reading Systems Framework across several languages however, the early years of word recognition may require somewhat different skills in different orthographies (McBride, 2017). Therefore, it is important that assessment of word reading ability include the various constructs (e.g., phonological processing, orthographic processing and morphological processing), as the research indicates they are thought to contribute to word reading ability given the unique characteristics of the language. The present study will examine these connections in Arabic.

Theories of skilled word reading suggest that when confronted with an unknown word, readers map a word’s letters and letter combinations to their sounds and use both orthographic and morphologic knowledge to access word meaning (Castles et al., 2018). This process is sometimes referred to as orthographic mapping and it plays a central role in word reading (Ehri, 2017). Readers are also able to gain access to word meaning from its spelling, without accessing phonology (Castles et al., 2018). When a word is known, direct access to meaning from spelling is more efficient (Share, 2008), but orthographic mapping (i.e., decoding), is used to access unknown words (Harm and Seidenberg, 2004). Depending on the features of the language, beginning readers may employ each of these processes to a different degree (Frost, 2012), whether the reading task is focused on accuracy or fluency, and where the student is in the stages of early reading development. Therefore, it is important to (1) understand how the distinctive characteristics of a language might influence word reading ability; (2) examine which processes explain variance in word reading ability (both accuracy and fluency); and (3) determine whether there are differences in the factors that predict word reading ability at different stages of reading development.

### 1.1. Characteristics of Arabic language

Three unique features of Arabic or Modern Standard Arabic (MSA) influence the development of word reading ability of young readers. First, Arabic is a diglossic language, in which its written form differs significantly from the different spoken forms of Arabic (Saiegh-Haddad, 2018). This results in young readers encountering a considerable number of unfamiliar words. Arabic diglossia also impacts children’s literacy skills at the letter name and sound level; for example, children are slowest to name letters that represent phonemes in MSA but not spoken Arabic (Asaad and Eviatar, 2014).

Second, the orthographic processing demands of written Arabic also present unique challenges to reading. Although the grapheme-phoneme correspondence of Arabic is generally transparent (Tibi and Kirby, 2018), there are several features that result in a much more complex, orthographically deep system (Tibi et al., 2021). Arabic has 28 distinct letters, 25 of which are consonants, 3 are long vowels. Most of the Arabic letters have more than one written form, depending on both the letter’s place in a word, as well as its connection to preceding or subsequent letters (Abu Rabia, 2007). Several letters are visually similar, differing primarily in the inclusion of dots (between one and three), placed either at the top or bottom of the letter. Additionally, the short vowels are either represented through diacritical markings, or omitted. The vowelized representation results in a largely shallow or transparent, orthography, whereas unvowelized text is considered a deep or opaque orthography (Taha, 2016). The effect of orthographic depth on word reading seems to be consistent in several languages. For example, Seymour et al. (2003) concluded that orthographic depth affects both word reading and nonword reading in the context of European languages. Moreover, the transparency of an orthography has been demonstrated to affect the amount of variance accounted for in reading performance by underlying cognitive processes such as phonological processing, memory, vocabulary, rapid naming, and nonverbal intelligence (Ziegler et al., 2010).

Finally, Arabic as a Semitic language, is characterized by a predominantly root and a derivational or inflectional pattern (Boudelaa, 2014) that gives a reader insight into the meaning of a word. The root provides the core meaning, and the derivation pattern provides both meaning as well as information about the grammatical category of the word (Boudelaa, 2014). Though these patterns can be useful, Arabic has sometimes been described as a morphologically opaque language because of the inconsistency with which the patterns apply (Bateson, 2003). There is limited research examining the role of morphological processing in Arabic word reading. However, results consistently indicate that morphological factors are important in reading across grade and reading ability levels (Abu-Rabia and Taha, 2006; Abu Rabia, 2007; Abu-Rabia and Abu-Rahmoun, 2012; Layes et al., 2017). Very few studies in Arabic have examined the role of morphological processing in very young readers, and, therefore, the role that morphological processing plays in early word reading development remains undecided.

These characteristics can present unique challenges to early readers and must be taken into consideration when examining the underlying cognitive processes of word reading. The hypothesis that young Arabic readers must map phonological information to corresponding orthographic information in order to recognize a word and that morphological processing enhances access to word meaning are both supported by existing research (Taha, 2013; Hassanein et al., 2021; Khoury-Metanis and Khateb, 2022). However, there are very few studies that have examined phonological processing, orthographic processing, and morphological processing simultaneously with a large sample of students across early grade levels. The research to date is also unclear as to whether different underlying processes account for variance in word reading abilities at different stages of reading development. This is a significant question, since it can inform a developmental instructional sequence, particularly for students who may struggle to acquire adequate word reading accuracy and fluency.

### 1.2. Examining processes that underlie word reading ability

As is the case in multiple languages, phonological processing was found to be a reliable predictor of both accurate and fluent word reading in Arabic. Phonological processing is the ability to hear and manipulate units of sound within spoken language (Harm and Seidenberg, 2004). Phonological processing consists of three constructs: (1) phonological awareness (PA), or the processing of and access to the sound structure of oral language; (2) phonological memory, or coding of information in a sound-based representation system for temporary storage; and (3) rapid automatized naming (RAN), or the rapid retrieval of phonological codes from permanent memory (Wagner and Torgesen, 1987). It is worth noting that the Rapid Automatized Naming (RAN), is considered as a phonological task by some authors but conceived as independent from phonology by others (for a review see Carioti et al., 2021, 2022). Several studies of phonological processing in Arabic include phonological processing tasks that are aligned to one or more of these constructs, but very few include tasks aligned to Wagner and Torgesen’s (1987) comprehensive definition of phonological processing. Additionally, this model of phonological processing guided the development of the Comprehensive Test of Phonological Processing (Wagner et al., 2013), which the current study used as a basis for the development of the Arabic measures of phonological processing skills suggested in the current study. All the tasks of phonological processing will be listed individually (e.g., elision, blending, segmenting, phoneme isolation, memory of digits, rapid naming of letters etc.) especially in data analysis.

Most studies examining the role of phonology with word reading rely on phonological awareness (PA) as opposed to phonological memory or rapid automatized naming (RAN) skills. Examples of PA skills are phoneme isolation (Asadi and Abu-Rabia, 2019; Gottardo et al., 2020), or elision (Elbeheri and Everatt, 2006). Across studies examining Arabic word reading accuracy, it is frequently reported that phonological awareness accounts for a substantial variance in performance (Asadi et al., 2017a; Schiff and Saiegh-Haddad, 2018; Asadi and Abu-Rabia, 2019; Gottardo et al., 2020). Studies that involve RAN assessments consistently find significant variance in RAN performance between dyslexic and typical readers, as well as strong relationships between RAN and measures of reading fluency (Ibrahim, 2013; Layes et al., 2017; Hassanein et al., 2022). Consistent with the research in several languages, there is evidence that the role of phonological awareness in word reading diminishes in later grades (Saiegh-Haddad, 2018). Very few studies include tasks that are aligned with the construct of phonological memory, and for those that do, the results are equivocal. For example, Taibah and Haynes (2011) found no relationship between measures of nonword repetition and digit span (both are considered to be phonological memory tasks) and reading performance of students in grades Kindergarten through 3. In contrast, Elbeheri and Everatt (2006) and Aboras et al. (2008) reported significant differences in performance on backwards digit span between strong readers and dyslexic students.

Orthographic processing is stored information in memory for the correct way to write words (Apel, 2011). According to Apel (2011), there are two components of orthographic processing (a) mental graphemic representations, developed through grapheme encoding, and (b) processing of orthographic rules that govern how speech is represented in writing in that language. Research across different languages confirms that orthographic processing is impacted by the visual complexity and the number of graphemes in the writing system, where more significant demands pose a challenge for beginning readers (Nag and Snowling, 2012; Chang et al., 2016).

Orthographic processing accounts for variance in both accurate and fluent Arabic word reading (Asadi et al., 2017b; Tibi et al., 2021; Khoury-Metanis and Khateb, 2022). Findings vary however, when examining whether vowelized texts support young readers’ word reading. For example, Abu-Rabia and Salfeety (2015) found that vowelized texts facilitated whereas Ibrahim (2013) reported that students read unvowelized words more quickly and accurately than vowelized versions of the same words. The current research suggests that orthographic processing is important at various stages in reading development, but to date we could locate only one study that examined the role of orthographic processing across different grade levels. In a cross-sectional path analysis, Asadi et al. (2017b) found that for measures of decoding (accuracy), orthographic processing played no role in first grade, but reached its maximum contribution in second grade. However, orthographic processing played a role across all grades when fluent word reading was the dependent variable.

Morphological processing is the ability to study the morphological units (the smallest unit with meaning) within words, including the explicit understanding of the relation between base words or roots, and related inflected and derived words (Apel and Lawrence, 2011). The morphological structure of Arabic plays a role in reading development (Abu Rabia, 2007; Saiegh-Haddad and Taha, 2017). Arabic morphology includes both derivational and inflectional structures (Abu Rabia, 2007). Derivational morphology is the creation of words from roots that are consonantal patterns. For example, the three-letter root k-t-b means “write.” A kitaab is a book, a katib, is a writer, and maktaba is a library, where the prefix m signifies a place, an example of inflectional morphology. Morphological processing has been assessed through a variety of tasks including having the examinee identify word roots and derive words from a given root (Abu Rabia, 2007; Hassanein et al., 2022).

Several studies suggest that morphological processing (MP) plays an important role in Arabic reading development (Asadi et al., 2017b; Vaknin-Nusbaum and Saiegh-Haddad, 2020; Tibi et al., 2021; Hassanein et al., 2022). Morphological processing is assessed in different ways, but most commonly through morpheme identification or word production tasks. MP emerges as a significant predictor of word reading, even when controlling for phonological processing and orthographic processing (Saiegh-Haddad and Taha, 2017; Hassanein et al., 2022). It has been theorized that MP is an important predictor of word reading for two primary reasons. First, MP has a reciprocal relationship with vocabulary, and vocabulary knowledge has been associated with strong word reading and comprehension skills in several languages (Liu and McBride-Chang, 2010; Wattad and Abu Rabia, 2020). Second, it has been theorized that the diglossic nature of Arabic requires young readers to rely on several sources of information to decode unknown, written words, including morphology (Saiegh-Haddad and Henkin-Roitfarb, 2014; Saiegh-Haddad and Taha, 2017; Vaknin-Nusbaum and Saiegh-Haddad, 2020; Wattad and Abu Rabia, 2020). Arabic has been described as a morphologically transparent orthography (Taha and Saiegh-Haddad, 2017), therefore, MP is an important skill for young readers.

### 1.3. Purpose of the current study

Despite a growing research to guide our knowledge of the variables that explain variation in Arabic word reading ability, the existing research is constrained by a number of issues. First, whereas many research look at only one or two early literacy variables (such as phonological processing or phonological and orthographic processing); very few studies look at these constructs collectively (Abu-Rabia and Abu-Rahmoun, 2012; Asadi et al., 2017b; Hassanein et al., 2022). Another limitation is that it remains unclear whether the contribution of the various processes might differ across the early grades when students are learning to read. It is worth noting that grade context is very important because reading instruction in Qatar is based on the grade they are in, therefore we expect that differences based on grade level are more likely than differences based on age. Additionally, teaching literacy skills in the early grades in Qatar follows the grade level, where the skills and the subskills are built to reflect Arabic language learning standards adopted by the Ministry of Education and Higher Education in the state (Morsi and Hassanein, 2023).

Given these limitations, it is essential to examine the relationship of phonological processing, orthographic processing, and morphological processing and to examine whether these relationships differ across the early grades. The purpose of the current study is to determine which early reading skills account for the most variance in word reading for young readers in grades 1 through 3, and to investigate whether different early reading skills account for variance when reading is measured by accuracy instead of fluency.

## 2. Materials and methods

### 2.1. Participants

In total, 1,098 students in grades 1 through 3 (443 of them girls) were chosen from eight public elementary schools in Doha, Qatar. Despite the fact that all data were gathered in Doha, the participating pupils come from nine different Arab countries, with 61.7% Qataris. First grade had a total of 268 students (Mage = 6.18 years, SDage = 0.46 years), (161 females), second grade had 483 students (Mage = 7.11 years, SDage = 0.48 years), (203 girls), and third grade had 347 students (Mage = 8.05 years, SDage = 0.4 years; 79 girls).

### 2.2. Measures

All constructs in this study were assessed using a new Arabic early literacy skills assessment, the TEALS (Hassanein et al., 2021, 2022). The TEALS consists of four subscales: (1) Phonological Processing, (2) Orthographic Processing, (3) Morphological Processing, and (4) Word Reading. Each subscale is described in more detail below.

#### 2.2.1. Phonological processing

The phonological processing subscale is comprised of 8 subtests: (1) elision, (2) blending, (3) segmenting, (4) phoneme isolation, (5) memory for digits, (6) nonword repetition, (7) rapid automatized naming-Digits, and (8) rapid automatized naming-Letters.

##### 2.2.1.1. Elision

This subtest includes 30 items in which the examinee hears a word and is asked to say the word while dropping a syllable (e.g., say “sunset” without the “sun”), or while dropping a phoneme (e.g., say “like” without the “l”). The score for this subtest is the number of correctly answered questions. Internal consistency for the Elision subtest with the current sample is 0.97.

##### 2.2.1.2. Blending

This subtest includes 30 items in which the examinee listens to separate parts (beginning at the syllable level, then progressing to the phoneme level) of a word and then blends them together to say the word. The score for this subtest is the number of correctly answered questions. Internal consistency for the Blending subtest with the current sample is 0.92.

##### 2.2.1.3. Segmenting

This subtest consists of 30 items in which the examinee listens to a word and then is asked to say parts of the word separately (beginning at the syllable level, then progressing to the phoneme level). The score for this subtest is the number of correctly answered questions. Internal consistency for the Segmenting subtest with the current sample is 0.95.

##### 2.2.1.4. Phoneme isolation

This subtest consists of 20 items in which the examinee hears a word, is told the number of phonemes within the word, and then is asked to identify one of those phonemes. The score for this subtest is the number of correctly answered questions. Internal consistency for the Phoneme Isolation subtest with the current sample is 0.95.

##### 2.2.1.5. Memory for digits

This subtest includes 20 items, in which the examinee listens to a string of digits, beginning with two and progressing to nine, and is asked to repeat them back. The score for this subtest is the number of correctly answered items, no partial credit is given. Internal consistency for the Memory for Digits subtest with the current sample is 0.82.

##### 2.2.1.6. Nonword repetition

In this task, the examinee hears words that are not real Arabic words and has to repeat them. This task includes 20 items beginning with one syllable words and progressing to words up to four syllables long. The score for this subtest is the number of correctly answered items. Internal consistency for the Nonword Repetition subtest with the current sample is 0.88.

##### 2.2.1.7. Rapid automatized naming digits/letters

In this task, the examinee is shown a 9 × 4 array of digits or letters, respectively. The rapid digit test was patterned from Landerl et al. (2019), and included only the digits 2, 3, 4, 6, 7, 8. Students are shown the array and are asked to name each item correctly. The total number of seconds it takes for the student to say all the items is recorded. The score for these subtests is the number of correct items named divided by the number of seconds.

#### 2.2.2. Orthographic processing

The orthographic processing subscale is comprised of three subtests: (1) letter/sound identification, (2) Identification of correctly spelled words (spelling) and (3) letter and letter sequence recognition (orthographic fluency).

##### 2.2.2.1. Letter name/sound identification

This subtest includes 30 items, in which students are shown a letter and first asked the letter name, then asked the letter sound. The score for this subtest is the number of correctly answered items. Internal consistency for the Letter Recognition subtest for the current sample is 0.96.

##### 2.2.2.2. Identification of correctly spelled words (spelling)

This subtest includes 20 items. Students are shown pairs of homophonic words and have to identify which is spelled correctly. The score for this subtest is the number of correctly answered items. Internal consistency for the current sample is 0.77.

##### 2.2.2.3. Letter and letter sequence recognition (orthographic fluency)

This subtest consists of 20 items and is patterned from the Feifer Assessment for Reading Orthographic Processing test (Feifer and Nader, 2015). Students are shown a stimulus card with a word on it for 1 s, then a second card that contains letters or letter sequences is shown. Only one letter or letter sequence will have been included in the word. The student is asked to point to the letter or letter sequence they saw on the word stimulus card. The score for this subtest is the number of correctly answered items. Internal consistency for this subtest for the current sample is 0.86.

#### 2.2.3. Morphological processing

The morphological processing subscale includes two subtests: (1) morpheme identification, and (2) word creation test.

##### 2.2.3.1. Morpheme identification

This subtest includes 20 items. For each item, the student is shown a word that is read to them. Then, the student sees a set of three additional words, two of which contain the same morpheme/root as the stimulus and one of which does not and has to identify which one of the three does not belong. The score for this subtest is the number of correctly answered items. Internal consistency for this subtest for the current sample is 0.91.

##### 2.2.3.2. Word creation test

This subtest includes 15 items. For each item, the student is given a root/morpheme. The student then must create three words that include that root. The student receives one point for each correct word formed, for a total of 45 possible points. Internal consistency for this subtest for the current sample is 0.97.

#### 2.2.4. Word reading

The word reading subscale includes two subtests: (1) untimed word reading and (2) word reading fluency. All lists of words were vowelized.

##### 2.2.4.1. Untimed word reading (reading accuracy)

Students are given a list of 30 vowelized words and asked to read them accurately. The score is the total number of correctly read words. Internal consistency for the current sample is 0.97.

##### 2.2.4.2. Word reading fluency

In this subtest, students are given 1 min to correctly read as many words from a list of 100 as possible. The score is the number of correctly read words. Internal consistency for the current sample is 0.97.

### 2.3. Procedures and data collection

We sought ethical clearance and approval of the research proposal from the Qatar University Institutional Review Board (IRB). The IRB have approved the research proposal. Next, we recruited six veteran teachers to participate in the process of data collection. Over the course of three, two-hour training sessions, the first author instructed the teachers on how to use TEALS (total of 6 h). Teacher were required to administer a pilot administration to three students after completing the training, and they were to note how long it took, how the kids responded to the test, and any other queries or issues they had with the assessment tool. To make sure they followed the prescribed administration procedures, the authors observed each teacher work with at least two students.

Then, teachers were assigned to the selected schools. To reduce distractions, tests were given to students in a calm 1:1 environment outside of the classroom. Students were provided with brief breaks, and since the students were young, the testing typically lasted about 50–60 min. Excel spreadsheets that follow the TEALS standard were used by teachers to record the findings. The first author then received the teachers’ data files and combined them into a single file. Data were collected between January through April 2022.

### 2.4. Data analysis

We conducted six separate multiple-regression analyses in order to determine the factors that explained the variance in word reading. First, students’ score on the untimed word-reading subtests (i.e., word reading accuracy) was computed as the dependent variable and then the subtest scores were entered as predictors. This analysis was conducted once more, but this time the dependent variable was a student’s performance on the timed word reading subtests (i.e., word reading fluency). We repeated these analyses for each grade level. We also conducted a one-way ANOVA to investigate differences in TEALS subtests by grade level. Multicollinearity was checked in two ways; using correlation coefficients and variance inflation factor (VIF) values. We transformed subtest scores to z scores before conducting the analyses. SPSS v.28 was used for conducting all analyses.

## 3. Results

Descriptive statistics are presented in Table 1. As can be seen in Table 1, mean scores across all subtests were lowest in first grade. Mean scores for blending, nonword repetition, RAN digits, RAN letters, morpheme identification, and word reading fluency were highest for third grade students. A one-way ANOVA indicated significant differences across all subtests except for word creation (see Table 2). *Post hoc* contrasts indicated that for each subtest, mean performance was statistically significant between first and second grade students, and between first and third grade students. For most subtests, the mean difference between second and third grade students was not statistically significant except for RAN-digits, RAN-letters, and segmenting.

**Table 1:** Means and standard deviations of subtests by grade level.

	First grade		Second grade		Third grade	
	M	SD	M	SD	M	SD
**Phonological processing**						
Elision	11.04	8.44	14.60	10.37	13.76	4.28
Blending	22.04	6.91	25.85	5.45	26.02	5.10
SEG	10.94	5.89	16.63	8.62	14.48	9.15
PI	14.17	6.30	16.71	4.67	16.55	5.12
MD	8.21	3.05	10.16	2.74	10.11	2.33
NWR	15.68	4.65	18.28	2.75	18.41	2.35
RAN-D	1.10	0.36	1.34	0.31	1.50	0.42
RAN-L	0.84	0.33	0.98	0.29	1.04	0.38
**Morphological processing**						
MI	11.66	6.00	13.62	5.70	13.86	4.70
WC	10.13	8.82	11.29	9.76	10.93	10.30
**Orthographic processing**						
LI	24.34	7.53	27.52	5.18	26.97	6.03
OF	11.87	5.04	14.05	4.62	13.36	4.28
Spelling	13.57	3.73	14.81	4.15	14.40	3.07
**Word reading**						
WRA	12.12	10.18	17.24	9.75	16.72	9.40
WRF	14.65	12.41	19.87	12.79	21.14	16.28

SEG, segmentation; PI, phoneme Isolation; MD, Memory of Digits; NWR, Non-word Repetition; RAND, Rapid Atomized Naming Digits; RANL, Rapid Atomized Naming Letters; MI, Morpheme Identification; WC, Word Creation; LI, Letter/Sound Identification; OF, Orthographic Fluency; WRA, Word Reading Accuracy; WRF, Word Reading Fluency.

**Table 2:** Summary of the one-way ANOVA of TEALS subscales scores by grade level.

Scale	Subscale	Grade level			*p*-value
		1	2	3	
		*n* = 268	*n* = 483	*n* = 347	
		Mean (SD)	Mean (SD)	Mean (SD)	
Phonological processing	Elision	36.8 (28.2)	48.7 (34.6)	45.9 (36.7)	<0.001
	Blending	73.5 (23.0)	86.2 (18.2)	86.7 (17.0)	<0.001
	SEG	36.5 (19.6)	55.4 (28.7)	48.3 (30.5)	<0.001
	PI	70.8 (31.5)	83.6 (23.3)	82.8 (25.6)	<0.001
	MD	41.1 (15.3)	50.8 (13.7)	50.6 (11.7)	<0.001
	NWR	78.4 (23.2)	91.4 (13.8)	92.1 (11.8)	<0.001
	RAN-D	1.10 (0.36)	1.34 (0.31)	1.50 (0.42)	<0.001
	RAN-L	0.85 (0.33)	0.99 (0.30)	1.05 (0.39)	<0.001
Morphological processing	MI	58.3 (30.0)	68.1 (28.5)	69.3 (23.5)	<0.001
	WC	22.5 (19.6)	25.1 (21.7)	24.3 (22.9)	0.537
Orthographic processing	LI	81.1 (25.1)	91.8 (17.3)	89.9 (20.1)	<0.001
	OF	59.3 (25.2)	70.2 (23.1)	66.8 (21.4)	<0.001
	Spelling	67.8 (18.6)	74.3 (20.8)	72.0 (15.4)	<0.001
Word Reading	WRA	40.4 (33.9)	57.5 (32.5)	55.7 (31.3)	<0.001
	WRF	20.0 (17.0)	27.2 (17.5)	29.0 (22.3)	<0.001

Multicollinearity was checked in two ways; using correlation coefficients and variance inflation factor (VIF) values. All correlation coefficients among the subtests (predictors) for each grade level (see Tables 3–5) have values less than 0.8 indicating that the predictors are not multicollinear. In addition, the multicollinearity assumption was checked using the variance inflation factor (VIF). All VIF values were less than 5 which indicates that the predictors are not multicollinear.

**Table 3:** Pearson’s linear correlation coefficients among the subtests of TEALS grade 1.

Subtest	Elision	Blending	SEG	PI	MD	NWR	RAND	RANL	MI	WC	LI	OF
Blending	0.706^**^	--										
SEG	0.674^**^	0.579^**^	--									
PI	0.630^**^	0.610^**^	0.636^**^	--								
MD	0.512^**^	0.621^**^	0.367^**^	0.577^**^	--							
NWR	0.340^**^	0.595^**^	0.302^**^	0.598^**^	0.759^**^	--						
RAND	0.478^**^	0.548^**^	0.405^**^	0.660^**^	0.679^**^	0.588^**^	--					
RANL	0.604^**^	0.574^**^	0.446^**^	0.677^**^	0.661^**^	0.546^**^	0.852^**^	--				
MI	0.522^**^	0.604^**^	0.467^**^	0.713^**^	0.674^**^	0.717^**^	0.541^**^	0.596^**^	--			
WC	0.452^**^	0.420^**^	0.598^**^	0.672^**^	0.404^**^	0.429^**^	0.526^**^	0.541^**^	0.570^**^	--		
LI	0.448^**^	0.558^**^	0.468^**^	0.722^**^	0.543^**^	0.599^**^	0.648^**^	0.678^**^	0.633^**^	0.536^**^	--	
OF	0.620^**^	0.582^**^	0.555^**^	0.716^**^	0.572^**^	0.541^**^	0.572^**^	0.676^**^	0.743^**^	0.625^**^	0.657^**^	--
Spelling	0.464^**^	0.514^**^	0.328^**^	0.480^**^	0.462^**^	0.430^**^	0.435^**^	0.482^**^	0.573^**^	0.394^**^	0.540^**^	0.692^**^

* = *p* < 0.05; ** = *p* < 0.01.

**Table 4:** Pearson’s linear correlation coefficients among the subtests of TEALS grade 2.

Subtest	Elision	Blending	SEG	PI	MD	NWR	RAND	RANL	MI	WC	LI	OF
Blending	0.475^**^	--										
SEG	0.378^**^	0.524^**^	--									
PI	0.608^**^	0.532^**^	0.458^**^	--								
MD	0.490^**^	0.485^**^	0.451^**^	0.506^**^	--							
NWR	0.247^**^	0.466^**^	0.353^**^	0.464^**^	0.551^**^	--						
RAND	0.360^**^	0.234^**^	0.278^**^	0.392^**^	0.334^**^	0.367^**^	--					
RANL	0.612^**^	0.353^**^	0.346^**^	0.568^**^	0.446^**^	0.376^**^	0.724^**^	--				
MI	0.537^**^	0.430^**^	0.419^**^	0.539^**^	0.558^**^	0.520^**^	0.377^**^	0.505^**^	--			
WC	0.538^**^	0.145^**^	0.056	0.359^**^	0.258^**^	0.113^*^	0.223^**^	0.455^**^	0.410^**^	--		
LI	0.380^**^	0.382^**^	0.357^**^	0.567^**^	0.356^**^	0.443^**^	0.244^**^	0.413^**^	0.463^**^	0.339^**^	--	
OF	0.642^**^	0.540^**^	0.574^**^	0.575^**^	0.557^**^	0.481^**^	0.345^**^	0.480^**^	0.689^**^	0.391^**^	0.562^**^	--
Spelling	0.597^**^	0.486^**^	0.440^**^	0.477^**^	0.467^**^	0.293^**^	0.214^**^	0.413^**^	0.595^**^	0.423^**^	0.554^**^	0.776^**^

* = *p* < 0.05; ** = *p* < 0.01.

**Table 5:** Pearson’s linear correlation coefficients among the subtests of TEALS grade 3.

+Subtest	Elision	Blending	SEG	PI	MD	NWR	RAND	RANL	MI	WC	LI	OF
Blending	0.429^**^	--										
SEG	0.459^**^	0.510^**^	--									
PI	0.559^**^	0.527^**^	0.388^**^	--								
MD	0.361^**^	0.307^**^	0.264^**^	0.293^**^	--							
NWR	0.256^**^	0.455^**^	0.276^**^	0.369^**^	0.256^**^	--						
RAND	0.507^**^	0.234^**^	0.104	0.458^**^	0.262^**^	0.151^**^	--					
RANL	0.536^**^	0.255^**^	0.094	0.493^**^	0.271^**^	0.093	0.790^**^	--				
MI	0.401^**^	0.260^**^	0.343^**^	0.430^**^	0.255^**^	0.271^**^	0.267^**^	0.275^**^	--			
WC	0.434^**^	0.116^*^	−0.057	0.329^**^	0.051	0.000	0.508^**^	0.584^**^	0.238^**^	--		
LI	0.337^**^	0.333^**^	0.209^**^	0.566^**^	0.164^**^	0.259^**^	0.367^**^	0.377^**^	0.434^**^	0.375^**^	--	
OF	0.438^**^	0.378^**^	0.460^**^	0.424^**^	0.287^**^	0.355^**^	0.226^**^	0.182^**^	0.502^**^	0.160^**^	0.525^**^	--
Spelling	0.552^**^	0.330^**^	0.382^**^	0.444^**^	0.200^**^	0.313^**^	0.272^**^	0.257^**^	0.497^**^	0.359^**^	0.524^**^	0.705^**^

* = *p* < 0.05; ** = *p* < 0.01.

The untimed word reading subtest was then used as the dependent variable in a multiple regression analysis by grade level to determine predictors of word reading accuracy (see Table 3). At first grade the regression model explained 83.2% of the variance. Seven subtests, Blending, Segmenting, Phoneme Isolation, Rapid Naming Letters, Nonword Repetition, Spelling, and Orthographic Fluency were statistically significant predictors. Five of the seven tests are subscales of the Phonological Processing scale, and two are subscales of the Orthographic Processing scale. No subscales of the Morphological Processing scale are statistically significant predictors, although Word Creation approached significance (Table 6).

**Table 6:** Multiple regression results for individual subtest predictors of word reading accuracy.

Variables^**^	First grade			Second grade			Third grade		
	*b*	*SE*	*p*	*B*	*SE*	*P*	*b*	*SE*	*p*
(Constant)	−0.128	0.037	0.001^*^	0.002	0.025	0.923	0.096	0.044	
Elision	0.075	0.058	0.200	0.089	0.040	0.028^*^	0.132	0.056	0.018^*^
Blending	0.099	0.039	0.011^*^	0.028	0.036	0.057	0.037	0.060	0.533
SEG	0.266	0.060	0.001^*^	0.061	0.032	0.057	0.051	0.048	0.285
PI	0.094	0.044	0.035^*^	0.074	0.043	0.081	0.058	0.060	0.331
MD	0.006	0.044	0.896	0.042	0.035	0.223	0.109	0.051	0.035^*^
NWR	−0.092	0.036	0.011^*^	−0.111	0.043	0.010^*^	−0.040	0.065	0.542
RAN-D	0.078	0.060	0.195	0.044	0.045	0.335	0.080	0.058	0.171
RAN-L	0.163	0.061	0.008^*^	0.002	0.051	0.974	0.020	0.061	0.750
MI	0.003	0.046	0.941	0.048	0.036	0.185	0.181	0.055	0.001^*^
WC	0.083	0.043	0.053	0.054	0.031	0.085	0.154	0.049	0.002^*^
LI	0.006	0.036	0.867	0.211	0.040	0.001^*^	0.125	0.055	0.023^*^
OF	0.503	0.048	0.001^*^	0.362	0.050	0.001^*^	0.338	0.065	0.001^*^
Spelling	0.190	0.038	0.001^*^	0.152	0.038	0.001^*^	0.113	0.075	0.134

^*^p < 0.05, ^**^Z scores.

At second grade the regression model explained 71.5% of the variance. Five subtests, Letter Identification, Spelling, Orthographic Fluency, Elision, and Nonword Repetition were statistically significant predictors. Three of the five subtests are part of the Orthographic Processing scale, and the other two are part of the Phonological processing scale. No subtests from the Morphological Processing scale were statistically significant predictors. At third grade, the regression model explained 66.2% of the variance. Six subtests were statistically significant predictors. Two of the subtests are part of the Orthographic Processing scale, two subtests are part of the Phonological processing scale, and two are part of the Morphological Processing scale. Table 7 lists the significant predictors by construct across each of the grade levels for comparison.

**Table 7:** Significant predictors of word reading accuracy by grade level.

First grade	Second grade	Third grade
**Phonological processing**		
	Elision	Elision
Blending		
Segmenting		
Isolation		
Rapid naming letters		
Nonword repetition	Nonword repetition	
		Memory for digits
**Orthographic processing**		
Orthographic fluency	Orthographic fluency	Orthographic fluency
Spelling	Spelling	
	Letter/sound identification	Letter/sound identification
**Morphological processing**		
		Word creation
		Morpheme identification

Table 8 shows the results of the regression model when word reading fluency was used as the dependent variable. At first grade, the regression model explained 76.6% of the variance. Six subtests were significant predictors. Two of the subtests were part of the Phonological Processing scale, with one subtest tapping Phonological Memory, and the other RAN. Subtests from each of the three scales (Phonological Processing, Orthographic Processing, and Morphological Processing) were statistically significant. At second grade, the regression model explained 65.7% of the variance. All three subtests of the Orthographic Processing scale and five subtests from the Phonological processing scale were significant predictors. Word Creation, a subtest of the Morphological Processing scale, was also a significant predictor. At third grade, the regression model explained 61.3% of the variance. Again, subtests from all three scales were significant predictors, including both subtests of the Morphological Processing scale and four subtests of the Phonological processing scale (Elision, Phoneme Isolation, Rapid Naming Digits, and Rapid Naming Letters). Table 9 presents a summary of the constructs by grade level that accounted for significant variance.

**Table 8:** Multiple regression results for individual subtest predictors of word reading fluency.

Variables^**^	First grade			Second grade			Third grade		
	*b*	*SE*	*p*	*b*	*SE*	*p*	*b*	*SE*	*p*
(Constant)	0.112	0.038	0.004^*^	0.014	0.025	0.569	0.068	0.045	0.133
Elision	0.036	0.059	0.543	0.113	0.041	0.006^*^	0.157	0.057	0.006^*^
Blending	0.076	0.040	0.056	0.018	0.037	0.623	0.073	0.061	0.238
SEG	0.031	0.061	0.616	0.095	0.032	0.003^*^	0.076	0.049	0.119
PI	0.050	0.045	0.264	0.118	0.043	0.007^*^	0.134	0.061	0.029*
MD	0.180	0.045	0.264	0.001	0.035	0.975	0.030	0.053	0.575
NWR	−0.107	0.036	0.004^*^	−0.155	0.044	0.001^*^	−0.053	0.067	0.427
RAN-D	0.073	0.061	0.232	0.096	0.046	0.037^*^	0.118	0.060	0.048^*^
RAN-L	0.173	0.062	0.006^*^	0.031	0.051	0.543	0.152	0.063	0.017^*^
MI	0.106	0.047	0.024^*^	0.013	0.036	0.718	0.151	0.057	0.008^*^
WC	0.123	0.044	0.006^*^	0.220	0.032	0.001^*^	0.311	0.051	0.001^*^
LI	0.070	0.037	0.055	0.107	0.041	0.009^*^	0.002	0.057	0.974
OF	0.410	0.050	0.001^*^	0.330	0.051	0.001^*^	0.333	0.067	0.001^*^
Spelling	0.149	0.039	0.001^*^	0.122	0.039	0.002^*^	0.279	0.078	0.001^*^

^*^p < 0.05, ^**^Z scores.

**Table 9:** Significant predictors of word reading fluency by grade level.

First grade	Second grade	Third grade
**Phonological processing**		
	Elision	Elision
Nonword repetition	Nonword repetition	
Rapid naming letters		Rapid naming letters
	Rapid naming digits	Rapid naming digits
	Isolation	Isolation
	Segmenting	
**Orthographic processing**		
Orthographic fluency	Orthographic fluency	Orthographic fluency
Spelling	Spelling	Spelling
	Letter/sound identification	Letter/sound identification
**Morphological processing**		
Word creation	Word creation	Word creation
Morpheme identification		Morpheme identification

## 4. Discussion

This research sought to: (1) examine the relationship of phonological processing, orthographic processing and morphological processing with Arabic word reading accuracy and fluency and (2) determine whether the relationship differs across the early elementary grade levels. Our findings showed that predictors of word reading varied slightly depending on whether word reading was assessed using an accuracy or a fluency measure. Additionally, predictors of word reading varied by grade level.

### 4.1. Predictors of word reading accuracy

In first grade, several subscales of phonological processing and two measures of orthographic processing accounted for significant differences in word reading accuracy. For second grade students, nonword repetition and elision were the only two phonological processing subscales that accounted for significant variance and all three measures of orthographic processing accounted for variance. Morphological processing subscales did not account for statistically significant variance in first or second grades. In third grade, elision and memory for digits, word creation and morpheme identification, and letter/sound identification and orthographic fluency were significant predictors of word reading accuracy.

#### 4.1.1. Phonological processing

The pattern of findings in the current study is consistent with much of the research examining the role of phonological processing in early Arabic reading ability. Numerous studies (e.g., Elbeheri and Everatt, 2006; Ibrahim, 2013; Tibi and Kirby, 2018; Asadi and Abu-Rabia, 2019; Landerl et al., 2019; Saiegh-Haddad et al., 2020) have consistently found that phonological processing accounts for a significant variance in word and text reading skills. As anticipated, the less complex phonological tasks (e.g., blending, segmenting, isolation) accounted for differences in first grade, but elision, typically considered a more complex task, accounted for differences in second and third grade. Additionally, across all three grade levels, at least one measure of phonological memory accounted for unique variance; nonword repetition in first and second grades and memory for digits in third grade.

RAN has been less commonly included in studies examining predictors of Arabic word reading accuracy, and its role is unclear across studies. In the current study, RAN-letters accounted for unique variance only for first graders. Both Asadi and Khateb (2017) and Hassanein et al. (2022) found that RAN did not play a significant role in word reading for first or second graders. Taibah and Haynes (2011) reported that RAN accounted for variance in a word reading measure across all grades, kindergarten through third grade. However, their study only examined phonological processing and did not include measures of orthography or morphology.

#### 4.1.2. Orthographic processing

Across all grades, measures of orthographic processing accounted for unique variance. Numerous studies have demonstrated that orthographic processing can significantly explain variation in reading accuracy (Ibrahim et al., 2002; Elbeheri et al., 2011; Asadi et al., 2017b; Maroun et al., 2019; Tibi et al., 2021). Interestingly, for first and second grade students, orthographic processing accounted for unique variance in word reading accuracy but no measure of morphological processing did. It is likely that orthographic processing alone explained unique variance because morphological units are recognized through print (Verhoeven and Perfetti, 2011). Morphological units are claimed to be a component of Arabic orthographic processing since they rely on the recognition of orthographic patterns to determine the root words and their derivations (Saiegh-Haddad, 2018). To put it another way, a child’s understanding of Arabic morphemes may be used to recognize correctly spelt words (orthographic choice) and vice versa.

#### 4.1.3. Morphological processing

Morphological Processing was a statistically significant predictor across third grade word reading accuracy only in this study. Our findings are consistent with others who report that morphology is a strong predictor of reading accuracy and comprehension in later grades (Abu-Rabia and Abu-Rahmoun, 2012; Tibi et al., 2019). Morphological processing may not play an important role in predicting first and second graders’ reading accuracy because they may rely more heavily on letter recognition (orthography), and their knowledge of grapheme – phoneme correspondence (Saiegh-Haddad, 2018). It has also been suggested that the ability to identify root words in Arabic may first rely on orthographic processing rather than morphological processing (Taha and Saiegh-Haddad, 2017).

### 4.2. Predictors of word reading fluency

In first grade, two subscales of phonological processing, two measures of orthographic processing, and two measures of morphological processing explained significant differences in word reading fluency. For second grade students, nonword repetition, elision, RAN-digits, isolation, and segmenting tasks explained significant variance. All three measures of orthographic processing and word creation also explained unique variance in word reading fluency. In third grade, elision, RAN-letters, RAN-digits and phoneme isolation, all measures of orthographic processing and both measures of morphological processing, explained variance in word reading fluency.

#### 4.2.1. Phonological processing

As anticipated, measures of RAN played a much more prevalent role when word reading was measured by fluency rather than accuracy. RAN has not been assessed extensively in Arabic. However, recent studies by Gharaibeh et al. (2021) and Tibi and Kirby (2018) found that RAN was an important predictor of third grade students’ word reading fluency. In one of few studies examining the role of RAN in reading fluency for younger students, Hassanein et al. (2022) found that RAN accounted for unique variance for first and second graders. The ability to automatically name digits and letters has been found to be an important predictor of word reading fluency across languages with transparent and opaque orthographies (Georgiou et al., 2008). Arabic is considered both transparent and opaque, depending on whether it is written with or without vowels.

#### 4.2.2. Orthographic processing

Orthographic processing emerged as an important factor in explaining variance across all three grades. This finding is consistent with an emerging research base examining the role of orthographic processing with Arabic word reading ability, although most studies tend to rely on measures of word reading accuracy rather than fluency. For example, Khoury-Metanis and Khateb (2022) found that measures of orthographic processing accounted for as much as 57% of variance in kindergarteners word reading accuracy and fluency ability. It is important to note that one of the measures of orthographic processing used in this study was a fluency measure – it is possible that a student’s processing speed is the common underlying trait for the orthographic fluency measure, RAN, and word reading fluency.

#### 4.2.3. Morphological processing

In contrast to our findings on the limited role of morphological processing in predicting word reading accuracy, measures of MP were statistically significant across all three grade levels when word reading fluency was the dependent variable. The importance of morphological processing to Arabic reading was recently highlighted in research conducted by Saiegh-Haddad (2018), who reported that MP as measured in a student’s spoken version of Arabic accounted for variance in reading fluency even after controlling for phonological processing. Our findings underscore the importance of MP in fluent word reading.

This study represents an important contribution to our understanding of the applicability of the Reading Systems Framework (Perfetti and Stafura, 2014), and to our understanding of Arabic early literacy development. Our findings suggest that phonological, orthographic and morphological processing all play a role in word reading accuracy and fluency, and, therefore, attention to students’ development of these skills is warranted.

Additionally, accuracy and fluency predictors varied by grade, where the ability to manipulate phonemes by dropping a syllable or sound in a word (i.e., elision), was a strong predictor for reading accuracy and fluency in grade 3, the basic phonological skills (e.g., blending, segmenting and isolation) were strong predictors of reading accuracy for grade 1. In terms of instruction, this requires more attention to phonological skills in early grades.

The findings of the current study indicated that both phonological and morphological processing skills played a significant role in predicting reading accuracy and fluency. These results could support the association between phonology and orthography especially transparent orthography (Ehri, 2017). Generally, the results of the current study supports the theory that beginning Arabic readers must map orthographic information to corresponding phonological information to identify a word (Elbeheri et al., 2011; Asadi et al., 2017b). Arabic orthography could be defined as phonologically transparent where the grapheme represents the same phoneme or the phoneme represents the same grapheme consistently (Alshaboul et al., 2014).

While the current instruction approaches of Arabic in Qatar focus on orthographic system, additional attention to phonological approach is required. On the other hand, the role of morphological processing in predicting accuracy was evident only for grade 3 and not grade 1 and 2. This magnifies the importance of morphological processing in higher grades. However, in early grades, the role of orthographic processing seems to be more significant giving that the ability to identify root words in Arabic may first depend on orthographic processing rather than morphological processing. Moreover, using diacritics in both lists of words for accuracy and fluency subtest used in the current study might have facilitated this mapping between phonology and morphology. However, this might not be the case in higher grades where the use of diacritics is likely to diminish in texts. This should be considered in future research.

## 5. Limitations and implications for future research

The study has a number of limitations that should be considered before generalizing results. Firstly, this is a cross-sectional study that examines the predictors of early reading skills. Secondly, data were collected following the pandemic, and this may have impacted third grade reading performance, which was equal to that of second graders for this sample of students. Third grade students were in first grade when the COVID-19 pandemic led to school shut-downs, and in Qatar, they remained shut down for most of the 2020–2021 school year. This means that these students missed 1.5 school years during a critical time for supporting reading development. Performance on the assessment may have been impacted—as evidenced by mean scores for third grade students that were essentially equivalent to second grade students. Finally, this study focused on word reading as the dependent variable. Relationship between reading comprehension and word reading should be examined in future research.

## 6. Conclusion

In spite of these limitations, this study significantly advances our knowledge of how Arabic early literacy develops. The results show that word reading ability is influenced by phonological, orthographic, and morphological processing, hence it is important to focus on helping children improve these abilities. The relative impact of these factors as pupils advance in their reading development should be further determined by longitudinal investigations in future research. Further research is required to determine how much instruction can help students acquire these abilities and improve their word reading.

## Data availability statement

The raw data supporting the conclusions of this article will be made available by the authors, without undue reservation.

## Ethics statement

The studies involving human participants were reviewed and approved by RIB Qatar University. Written informed consent to participate in this study was provided by the participants’ legal guardian/next of kin.

## Author contributions

EH: conceptualization, methodology, data curation, and writing—review and editing. EJ: conceptualization and writing—original draft. SI: methodology and data curation. YA: methodology and data curation. All authors contributed to the article and approved the submitted version.

## Funding

This research was supported by the Qatar National Foundation, award number NPRP11S-1211-170087.

## Conflict of interest

The authors declare that the research was conducted in the absence of any commercial or financial relationships that could be construed as a potential conflict of interest.

## Publisher’s note

All claims expressed in this article are solely those of the authors and do not necessarily represent those of their affiliated organizations, or those of the publisher, the editors and the reviewers. Any product that may be evaluated in this article, or claim that may be made by its manufacturer, is not guaranteed or endorsed by the publisher.
